# Oscillation Control Algorithms for Resonant Sensors with Applications to Vibratory Gyroscopes

**DOI:** 10.3390/s90805952

**Published:** 2009-07-27

**Authors:** Sungsu Park, Chin-Woo Tan, Haedong Kim, Sung Kyung Hong

**Affiliations:** 1 Department of Aerospace Engineering, Sejong University, Seoul 143-747, Korea; 2 PINC Solutions, Berkeley, CA 94709, USA

**Keywords:** oscillation control, resonant sensor, vibratory gyroscope, averaging method, automatic gain control, phase-locked loop

## Abstract

We present two oscillation control algorithms for resonant sensors such as vibratory gyroscopes. One control algorithm tracks the resonant frequency of the resonator and the other algorithm tunes it to the specified resonant frequency by altering the resonator dynamics. Both algorithms maintain the specified amplitude of oscillations. The stability of each of the control systems is analyzed using the averaging method, and quantitative guidelines are given for selecting the control gains needed to achieve stability. The effects of displacement measurement noise on the accuracy of tracking and estimation of the resonant frequency are also analyzed. The proposed control algorithms are applied to two important problems in a vibratory gyroscope. The first is the leading-following resonator problem in the drive axis of MEMS dual-mass vibratory gyroscope where there is no mechanical linkage between the two proof-masses and the second is the on-line modal frequency matching problem in a general vibratory gyroscope. Simulation results demonstrate that the proposed control algorithms are effective. They ensure the proof-masses to oscillate in an anti-phase manner with the same resonant frequency and oscillation amplitude in a dual-mass gyroscope, and two modal frequencies to match in a general vibratory gyroscope.

## 1. Introduction

Robust operation of a resonant sensor, such as a vibratory gyroscope, requires the resonator to be driven at resonance with constant amplitude. However, both the amplitude and resonant frequency can vary due to environmental factors, such as changes in temperature or stiffness aging. Therefore, some form of oscillation control is needed to track the constantly changing resonant frequency or to tune it to a specified frequency while keeping the amplitude constant.

Automatic gain control (AGC) has generally been used to excite the resonator to track the reference amplitude. The application of AGC to the drive axis of a vibratory gyroscope is reported in [1], where velocity measurement is used to control the velocity amplitude to maintain the reference amplitude. It also presented stability analysis results and AGC performance, but did not provide the noise analysis. Typically the velocity sensing circuitry produces larger noise than the displacement sensing circuitry does. A phase-locked loop (PLL) has been also used to track a resonant frequency in [2], where stability and resolution analysis results of PLL frequency tracking system for MEMS fatigue testing are reported. However, it did not suggest a method to sustain the specified amplitude.

There have also been a few studies on controllers which, instead of tracking the resonant frequency, tune it to a specified frequency chosen by the designer. The advantages of this method are that it can maintain consistent performance, since the sensor can retain the dynamic characteristics regardless of environmental factors such as temperature changes, and simplify the signal processing loop that uses the resonant frequency as its carrier frequency. In the literature, only Lyapunov based adaptive control schemes for vibratory gyroscopes are reported to place the resonant frequency at a specified frequency [3,4].

This paper presents two algorithms for controlling the frequency and amplitude of oscillation. One control algorithm tracks the resonant frequency and the other algorithm tunes it to the specified resonant frequency by altering the resonator dynamics. Both algorithms maintain the specified amplitude of oscillations. In the first algorithm, AGC and PLL structures are used to control the amplitude and track the resonant frequency. The displacement measurement is used to avoid using noisy velocity measurement. The second algorithm is similar to [3,4], in that the reference frequency is chosen and then resonant frequency is adapted to it. However, it is different in some ways. First, our algorithm modifies the PLL structure to tune the resonant frequency and uses conventional AGC to regulate the amplitude, making it simpler than an adaptive control approach. Second, our algorithm can adapt only the frequency to the reference and set amplitude free rather than regulating it, which makes it applicable to mode tuning on the sense axis of a vibratory gyroscope, whereas an adaptive control approach cannot.

The averaging method is used to analyze the stability of the entire feedback system and the effects of the displacement measurement noise on tracking and estimation of the resonant frequency. The proposed control algorithms are applied to two important problems in a vibratory gyroscope to evaluate their performance. The first one is the leading-following resonator problem in the drive axis of a MEMS dual-mass vibratory gyroscope without a mechanical linkage between the two proof-masses. Adopting the proposed control algorithms enables the implementation of the following resonator which precisely traces the oscillation pattern of the leading resonator. The second one is the on-line modal frequency matching problem in a general vibratory gyroscope. Applying the first algorithm on the drive axis and the second algorithm on the sense axis enables the resonant frequencies of the drive and sense axes to be matched precisely, which significantly improves the gyroscope performance.

## 2. Frequency Tracking and Amplitude Control

The equation of motion of a resonator is modeled as a spring-mass-damper system and described as a second-order differential equation as shown in (1):

(1) $$\ddot{x}+d\dot{x}+\omega_{n}^{2}x=f$$

where *x* is the displacement of resonator, *d* is the normalized damping coefficient, *ω_n_* is the natural frequency, and *f* is control input.

### 2.1. Control Algorithm

The control input used to excite the resonator at constant amplitude, *X*_0_, while tracking the resonant frequency of the resonator is given by:

(2) f=A cos θ

where *A* is the magnitude of the input and *θ* is the instantaneous phase of the input. The magnitude should be chosen to make the difference between the measured and the specified amplitude, *X*_0_, equal to zero. Therefore, *A* should have an AGC form. On the other hand, cos*θ* should have a PLL form because it should recognize the resonance when the difference in phase angle between a control input and a displacement is 90°.

The AGC is composed of a rectifier, a low-pass filter, and a comparator [1]. However, in order to precisely maintain the specified amplitude, *X*_0_, we employ a proportional-integral control in the AGC. The proposed AGC in this paper is as follows:

(3) $$\begin{array}{l} A=K_{p}\;\left(X_{0}-r\right)+B\\ \dot{B}=K_{I}\;\left(X_{0}-r\right)\\ \dot{r}=\lambda_{a}\left(\frac{\pi }{2}|x+n_{p}|-r\right) \end{array}$$

where *K_p_* is the proportional control gain; *K_I_* is the integral control gain; *r* is an estimation of the amplitude of *x*, which is the low-pass filtered value of the absolute value of *x*; and *λ_a_* is the corner frequency of the low-pass filter. *n_p_* is the displacement measurement noise and is assumed to be a white Gaussian noise with zero mean and power spectral density (PSD) of $\sigma_{p}^{2}\;\left[m^{2}/Hz\right]$.

**Figure 1.**
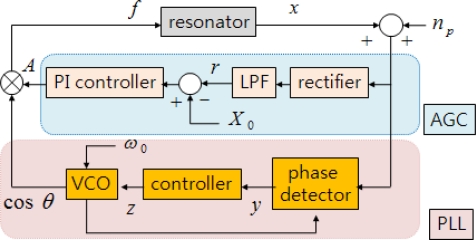
Block diagram of frequency tracking and amplitude control.

A PLL is composed of a phase detector, a PLL controller, and a voltage-controlled oscillator (VCO). Each can be written as follows [2]:

(4) $$\begin{array}{l} \omega \equiv \dot{\theta }=\omega_{0}+K_{v}\;z\\ \dot{z}=K_{z}\;y\\ \dot{y}=\lambda_{p}\left(\left(x+n_{p}\right)\;\text{cos}\;\theta -y\right) \end{array}$$

where cos*θ* is the output of the VCO. The instantaneous phase angle, *θ*, is the integral of the instantaneous frequency *ω*, which is the sum of a proportional value of the control voltage of VCO, *z*, and the free oscillation frequency, *ω*_0_. The VCO control voltage, *z*, is the integral of the output of the phase detector *y*, which is the calculated difference in phase between the output of the VCO and the displacement, and is given as the low-pass filtered value of cos*θ* multiplied by the displacement. In (4), *K_v_* and *K_z_* are the gains of VCO and integral control, respectively, and *λ_p_* is the corner frequency of the low-pass filter. Figure 1 shows a block diagram of the frequency tracking and amplitude control composed of an AGC and a PLL.

### 2.2. Stability Analysis

This section follows the formulation in [1,2] and employs the averaging method to analyze the stability of the feedback system depicted in Figure 1 with respect to the design parameters: the control gains *K_p_* and *K_I_* of AGC, the control gains *K_v_*, *K_z_* of PLL, and corner frequencies *λ_a_*, *λ_p_* of the low-pass filter.

Transforming of the displacement and velocity, *x* and *ẋ*, to the amplitude, *a*, and the phase angle, *ϕ*, and applying them to the feedback system composed of (1), and controllers (2), (3), and (4) yield:

(5) $$\begin{array}{l} \dot{a}=-\frac{1}{\omega_{0}+K_{v}\;z}[\left(K_{p}\;\left(X_{0}-r\right)+B\right)\;\text{sin}(\theta +\phi )\;\text{cos}\;\theta \\ \;+a\left\{K_{v}K_{z}\;y+d\left(\omega_{0}+K_{v}\;z\right)\right\}{\text{sin}}^{2}\;(\theta +\phi )\\ \;+a\left\{{\left(\omega_{0}+K_{v}\;z\right)}^{2}-\omega_{n}^{2}\right\}\text{sin}(\theta +\phi )\;\text{cos}(\theta +\phi )] \end{array}$$

(6) $$\begin{array}{l} \dot{\phi }=-\frac{1}{\omega_{0}+K_{v}\;z}[\frac{1}{a}\left(K_{p}\left(X_{0}-r\right)+B\right)\;\text{cos}(\theta +\phi )\;\text{cos}\;\theta \\ \;+\left\{K_{v}K_{z}\;y+d\left(\omega_{0}+K_{v}z\right)\right\}\text{sin}(\theta +\phi )\;\text{cos}(\theta +\phi )\\ \;+\left\{{\left(\omega_{0}+K_{v}\;z\right)}^{2}-\omega_{n}^{2}\right\}{\text{cos}}^{2}\;(\theta +\phi )] \end{array}$$

(7) $$\begin{array}{l} \dot{B}=K_{I}\;\left(X_{0}-r\right)\\ \dot{r}\approx \lambda_{a}\left(\frac{\pi }{2}|a\;\text{cos}(\theta +\phi )|-r\right)+\lambda_{a}\frac{\pi }{2}|n_{p}| \end{array}$$

(8) $$\begin{array}{l} \dot{z}=K_{z}\;y\\ \dot{y}=\lambda_{p}\left\{a\;\text{cos}(\theta +\phi )\;\text{cos}\;\theta -y\right\}+\lambda_{p}\;n_{p}\;\text{cos}\;\theta \end{array}$$

In (7), *r* denotes the estimate of the amplitude of *x*, so *r* ≥ 0, and the noise term can be separated by using |*x* + *n_p_*| ≤ |*x*| + |*n_p_*|. The mean value of |*n_p_*|, which is denoted as *n*_0_, is calculated as:

(9) $$n_{0}=\frac{{2\sigma }_{p}}{\sqrt{2\pi }}$$

If *m_p_* is defined as:

(10) $$m_{p}=|n_{p}|-n_{0}$$

the mean value of *m_p_* is zero, and the PSD of *m_p_* and the correlation of *m_p_* and *n_p_* are given by:

(11) $$\begin{array}{l} {\bar{m}}_{p}^{2}={\bar{n}}_{p}^{2}-n_{0}^{2}\approx {0.36\sigma }_{p}^{2}\\ \overset{¯}{m_{p}n_{p}}=\sigma_{p}^{2} \end{array}$$

where the bar denotes the stochastic expectation. With (10), (7) can be rewritten as:

(12) $$\dot{r}=\lambda_{a}\left(\frac{\pi }{2}(|a\;\text{cos}(\theta +\phi )|+n_{0})-r\right)+\lambda_{a}\frac{\pi }{2}m_{p}$$

Equations (5)–(8) are simplified to a general stochastic nonlinear state space equation as follows.

(13) $${\dot{x}}_{1}=f_{1}\left(x_{1}\right)+g_{1}w$$

where:

(14) $$\begin{array}{l} x_{1}={\left[a\;\phi \;B\;r\;z\;y\right]}^{T},\;w={\left[m_{p}\;n_{p}\right]}^{T}\\ g_{1}={\left[\begin{array}{cccccc} 0 & 0 & 0 & \lambda_{a}\pi /2 & 0 & 0\\ 0 & 0 & 0 & 0 & 0 & \lambda_{p}\;\text{cos}\;\theta \end{array}\right]}^{T} \end{array}$$

A first-order approximation of the Taylor series expansion of the nonlinear function **f_1_(x_1_)** in (13) about the mean, **x̄_1_**, yields a time update of expectation as follows:

(15) $${\dot{\bar{x}}}_{1}=f_{1}\left({\bar{x}}_{1}\right)$$

Since *θ* changes much faster than other variables, the averaging method can be applied to (15), and the averaged dynamic equations are obtained as follows:

(16) $${\dot{\bar{x}}}_{1\mathrm{av}}=f_{1\mathrm{av}}\;\left({\bar{x}}_{1\mathrm{av}}\right)$$

where subscript, *av*, denotes the averaged value. Equation (16) can be written in detail as follows.

$$\begin{array}{l} {\dot{\bar{a}}}_{\mathrm{av}}=-\frac{1}{\omega_{0}+K_{v}\bar{z}}[\frac{1}{2}\left(K_{p}\left(X_{0}-{\bar{r}}_{\mathrm{av}}\right)+{\bar{B}}_{\mathrm{av}}\right)\text{sin}\;{\bar{\phi }}_{\mathrm{av}}\\ \;+\frac{{\bar{a}}_{\mathrm{av}}}{2}\left\{K_{v}K_{z}{\bar{y}}_{\mathrm{av}}+d\left(\omega_{0}+K_{v}{\bar{z}}_{\mathrm{av}}\right)\right\}] \end{array}$$

$$\begin{array}{l} {\dot{\bar{\phi }}}_{\mathrm{av}}=-\frac{1}{\omega_{0}+K_{v}{\bar{z}}_{\mathrm{av}}}[\frac{1}{{2\bar{a}}_{\mathrm{av}}}\left(K_{p}\left(X_{0}-{\bar{r}}_{\mathrm{av}}\right)+{\bar{B}}_{\mathrm{av}}\right)\text{cos}\;{\bar{\phi }}_{\mathrm{av}}\\ \;+\frac{1}{2}\left\{{\left(\omega_{0}+K_{v}{\bar{z}}_{\mathrm{av}}\right)}^{2}-\omega_{n}^{2}\right\}] \end{array}$$

$$\begin{array}{l} {\dot{\bar{B}}}_{\mathrm{av}}=K_{I}\;(X_{0}-{\bar{r}}_{\mathrm{av}})\\ {\dot{\bar{r}}}_{\mathrm{av}}=\lambda_{a}\left({\bar{a}}_{\mathrm{av}}+\frac{\pi }{2}n_{0}-{\bar{r}}_{\mathrm{av}}\right) \end{array}$$

$$\begin{array}{l} {\dot{\bar{z}}}_{\mathrm{av}}=K_{z}{\bar{y}}_{\mathrm{av}}\\ {\dot{\bar{y}}}_{\mathrm{av}}=\lambda_{p}\left(\frac{1}{2}{\bar{a}}_{\mathrm{av}}\;\text{cos}\;{\bar{\phi }}_{\mathrm{av}}-{\bar{y}}_{\mathrm{av}}\right) \end{array}$$

An equilibrium point of (16) is:

$${\bar{r}}_{0}=X_{0},\;{\bar{a}}_{0}=X_{0}-\frac{\pi }{2}n_{0},\;{\bar{\phi }}_{0}=-\frac{\pi }{2},\;{\bar{B}}_{0}={\bar{a}}_{0}d\omega_{n},\;{\bar{z}}_{0}=\frac{\omega_{n}-\omega_{0}}{K_{v}},\;{\bar{y}}_{0}=0$$

The Jacobian matrix of the averaged system (16) at the equilibrium point is:

(17) $$F_{1}\equiv \frac{\partial f_{1\mathrm{av}}}{{\partial x}_{1}}=\left[\begin{array}{cccccc} -\frac{d}{2} & 0 & \frac{1}{{2\omega }_{n}} & -\frac{K_{p}}{{2\omega }_{n}} & -\frac{{\bar{a}}_{0}{\mathrm{dK}}_{v}}{{2\omega }_{n}} & -\frac{{\bar{a}}_{0}K_{v}K_{z}}{{2\omega }_{n}}\\ 0 & -\frac{d}{2} & 0 & 0 & -K_{v} & 0\\ 0 & 0 & 0 & -K_{I} & 0 & 0\\ \lambda_{a} & 0 & 0 & -\lambda_{a} & 0 & 0\\ 0 & 0 & 0 & 0 & 0 & K_{z}\\ 0 & \frac{\lambda_{p}{\bar{a}}_{0}}{2} & 0 & 0 & 0 & -\lambda_{p} \end{array}\right]$$

The conditions for the above Jacobian matrix to be stable are:

(18) $$\left(d+\frac{K_{p}}{\omega_{n}}\right)\;\left(\frac{d}{2}+\lambda_{a}\right)>\frac{K_{I}}{\omega_{n}},\;d\;\left(\frac{d}{2}+\lambda_{p}\right)>{\bar{a}}_{0}K_{v}K_{z}$$

Equation (18) is the stability criteria of the control system and can be used for selecting control parameters for achieving stability. The left-hand side of (18) consists of the design parameters related only to AGC, while the right-hand side consists of the design parameters related only to PLL. Therefore, we can design each controller separately. If the control system is stable, *θ̇* = *ω_n_* is achieved and the excitation frequency tracks the resonant frequency, *ω_n_*. In addition, since *ā*_0_ = *X*_0_ − *n*_0_π/2, the amplitude converges to the spcified value, *X*_0_, with a small deviation due to the displacement measurement noise.

### 2.3. Resolution Analysis

As seen in the previous section, the displacement measurement noise not only causes errors in the amplitude control, but also affects the resolution of the resonant frequency tracking. Applying the covariance propagation equation, this section describes the effects of displacement measurement noise on the resolution of the resonant frequency tracking controller. The covariance propagation equation of (13) is defined as:

(19) $${\dot{P}}_{1}=\frac{d}{\mathrm{dt}}\left[\overset{¯}{\left(x_{1}-{\bar{x}}_{1}\right){\left(x_{1}-{\bar{x}}_{1}\right)}^{T}}\right]$$

Expanding above equation using the Taylor series at the mean, **x̄_1_**, and obtaining a first-order approximation yields the covariance propagation equation as follows [3]:

(20) $${\dot{P}}_{1}=P_{1}{\left(\frac{{\partial f}_{1}}{{\partial x}_{1}}\right)}^{T}+\left(\frac{{\partial f}_{1}}{{\partial x}_{1}}\right)\;P_{1}+g_{1}(\bar{x}){Sg}_{1}{\left(\bar{x}\right)}^{T}$$

where the PSD of measurement noise vector, *S*, is given by:

(21) $$S=\left[\begin{array}{cc} {0.36\sigma }_{p}^{2} & \sigma_{p}^{2}\\ \sigma_{p}^{2} & \sigma_{p}^{2} \end{array}\right]$$

Applying the averaging method to (20) yields the covariance equations for (13) at steady state as:

(22) $$0=P_{1}F_{1}^{T}+F_{1}P_{1}+Q_{1}$$

where *F*_1_ is defined in (17) and:

$$Q_{1}=\mathrm{diag}\left\{0,\;0,\;0,\;{\left(\lambda_{a}\pi /2\right)}^{2}\left(0.36\sigma_{p}^{2}\right),\;0,\;\lambda_{p}^{2}\sigma_{p}^{2}/2\right\}\;$$

The standard deviation, which is the averaged resolution of excitation frequency in the frequency tracking control, is derived as:

(23) $$\sigma_{1}=K_{v}\sqrt{P_{1}(5,5)}$$

where *P*_1_(5,5) denote (5,5) terms of the numerical solution of (22).

## 3. Frequency Tuning and Amplitude Control

### 3.1. Control Algorithm

**Figure 2.**
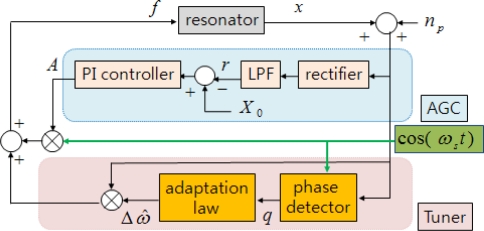
Block diagram of frequency tuning and amplitude control.

The frequency tuning control differs from the frequency tracking control in that it adjusts the dynamic characteristics of the resonator to match to a resonant frequency specified by the designer, instead of its own resonant frequency. This requires controlling the resonant frequency through the displacement feedback. Adopting the specified resonant frequency, *ω_s_*, Equation (1) is rewritten as follows:

(24) $$\ddot{x}+d\dot{x}+\omega_{s}^{2}x+\Delta \omega \;x=f$$

where $\Delta \omega =\omega_{n}^{2}-\omega_{s}^{2}$ is the difference between the actual and the specified resonant frequencies. The objective of the frequency tuning control is to compensate this value. Therefore, the control input is consisted of a part that excites the system at the specified resonant frequency, *ω_s_*, and another part that compensates the difference in the resonant frequency through the displacement feedback. This is given by:

(25) $$f=A\;\text{cos}\left(\omega_{s}\;t\right)+\Delta \hat{\omega }\;\left(x+n_{p}\right)$$

where *A* is in AGC form, identical to (3), because it is an amplitude control that maintains the amplitude of the displacement at the specified value. Here Δ*ω̂* is an estimate of Δ*ω*, and *n_p_* is the displacement measurement noise. The adaptation law of Δ*ω̂* is derived from the fact that the phase difference between the control input and the displacement is −90° when Δ*ω̂=* Δ*ω*. The proposed adaptation law in this paper comprises the phase detector and the integral controller and is given by:

(26) $$\begin{array}{l} \Delta \dot{\hat{\omega }}=K_{\omega }\;q\\ \dot{q}=\lambda_{q}\left(\left(x+n_{p}\right)\;\text{cos}\left(\omega_{s}\;t\right)-q\right) \end{array}$$

where *q* is the low-pass filtered signal of the control input multiplied by the displacement, *K_ω_* is the integral gain, and *λ_q_* is the corner frequency of the low-pass filter. Figure 2 illustrates the block diagram of the frequency tuning and amplitude control.

### 3.2. Stability Analysis

Similarly to the previous section, transforming of the displacement and velocity, *x* and *ẋ*, to the amplitude, *a*, and the phase angle, *ϕ*, and applying them to the feedback system composed of (1), and controllers (3), (25), and (26) yields.

(27) $$\begin{array}{l} \dot{a}=-\frac{1}{\omega_{s}}[\left(K_{p}\left(X_{0}-r\right)+B\right)\;\text{sin}\left(\omega_{s}\;t+\phi \right)\;\text{cos}\left(\omega_{s}\;t\right)\\ \;+\Delta \hat{\omega }n_{p}\;\text{sin}\left(\omega_{s}\;t+\phi \right)+\mathrm{ad}\omega_{s}\;{\text{sin}}^{2}\;\left(\omega_{s}\;t+\phi \right)\\ \;-a\Delta \tilde{\omega }\text{sin}\left(\omega_{s}\;t+\phi \right)\;\text{cos}\left(\omega_{s}\;t+\phi \right)] \end{array}$$

(28) $$\begin{array}{l} \dot{\phi }=-\frac{1}{\omega_{s}}[\frac{1}{a}\left(K_{p}\left(X_{0}-r\right)+B\right)\;\text{cos}\left(\omega_{s}\;t+\phi \right)\;\text{cos}\left(\omega_{s}\;t\right)\\ \;+\frac{1}{a}\Delta \hat{\omega }n_{p}\;\text{cos}\left(\omega_{s}\;t+\phi \right)+{d\omega }_{s}\;\text{sin}\left(\omega_{s}\;t+\phi \right)\;\text{cos}\left(\omega_{s}\;t+\phi \right)\\ \;-\Delta \tilde{\omega }{\text{cos}}^{2}\;\left(\omega_{s}\;t+\phi \right)] \end{array}$$

(29) $$\begin{array}{l} \dot{B}=K_{I}\;\left(X_{0}-r\right)\\ \dot{r}\approx \lambda_{a}\left(\frac{\pi }{2}\left(|a\;\text{cos}\left(\omega_{s}\;t+\phi \right)|+n_{0}\right)-r\right)+\lambda_{a}\;\frac{\pi }{2}\;m_{p} \end{array}$$

(30) $$\begin{array}{l} \Delta \dot{\tilde{\omega }}=-K_{\omega }\;q\\ \dot{q}=\lambda_{q}\left\{a\;\text{cos}\left(\omega_{s}\;t+\phi \right)\;\text{cos}\left(\omega_{s}\;t\right)-q\right\}+\lambda_{q}\;n_{p}\;\text{cos}\left(\omega_{s}\;t\right) \end{array}$$

where (29) is identical to (7), and Δ*ω̃* = Δ*ω* − Δ*ω̂* denotes the estimation error. Equations (27) (30) are simplified to a general stochastic nonlinear equation as:

(31) $${\dot{x}}_{2}=f_{2}\left(x_{2}\right)+g_{2}w$$

where:

(32) $$\begin{array}{l} x_{2}={\left[a\;\phi \;B\;r\;\Delta \tilde{\omega }\;q\right]}^{T},\;w={\left[m_{p}\;n_{p}\right]}^{T}\\ g_{2}={\left[\begin{array}{cccccc} 0 & 0 & 0 & \lambda_{a}\pi /2 & 0 & 0\\ \Delta \hat{\omega }\text{sin}\left(\omega_{s}\;t+\phi \right) & \Delta \hat{\omega }\text{cos}\left(\omega_{s}\;t+\phi \right)/a & 0 & 0 & 0 & \lambda_{q}\;\text{cos}\left(\omega_{s}\;t+\phi \right) \end{array}\right]}^{T} \end{array}$$

Similarly to the previous section, an approximated time update of expectation of (31) is obtained as follows:

(33) $${\dot{\bar{x}}}_{2}=f_{2}\left({\bar{x}}_{2}\right)$$

Applying the same method yields the nonlinear averaged dynamics of (33) as:

(34) $${\dot{\bar{x}}}_{2\mathrm{av}}=f_{2\mathrm{av}}\left({\bar{x}}_{2\mathrm{av}}\right)$$

or in detailed forms as:

$$\begin{array}{l} {\dot{\bar{a}}}_{\mathrm{av}}=-\frac{1}{2\omega_{s}}\left[\left(K_{p}\;\left(X_{0}-{\bar{r}}_{\mathrm{av}}\right)+{\bar{B}}_{\mathrm{av}}\right)\;\text{sin}\;{\bar{\phi }}_{\mathrm{av}}+{d\omega }_{s}\;{\bar{a}}_{\mathrm{av}}\right]\\ {\dot{\bar{\phi }}}_{\mathrm{av}}=-\frac{1}{{2\omega }_{s}}\left[\frac{1}{{\bar{a}}_{\mathrm{av}}}\left(K_{p}\;\left(X_{0}-{\bar{r}}_{\mathrm{av}}\right)+{\bar{B}}_{\mathrm{av}}\right)\;\text{cos}\;{\bar{\phi }}_{\mathrm{av}}-\Delta {\bar{\tilde{\omega }}}_{\mathrm{av}}\right] \end{array}$$

$$\begin{array}{l} {\dot{\bar{B}}}_{\mathrm{av}}=K_{I}\;\left(X_{0}-{\bar{r}}_{\mathrm{av}}\right)\\ {\dot{\bar{r}}}_{\mathrm{av}}=\lambda_{a}\left({\bar{a}}_{\mathrm{av}}+\frac{\pi }{2}n_{0}-{\bar{r}}_{\mathrm{av}}\right) \end{array}$$

$$\begin{array}{l} \Delta {\dot{\bar{\tilde{\omega }}}}_{\mathrm{av}}=-K_{\omega }\;{\bar{q}}_{\mathrm{av}}\\ {\dot{\bar{q}}}_{\mathrm{av}}=\lambda_{q}\left(\frac{1}{2}{\bar{a}}_{\mathrm{av}}\;\text{cos}{\bar{\phi }}_{\mathrm{av}}-{\bar{q}}_{\mathrm{av}}\right) \end{array}$$

An equilibrium point of (34) is:

$${\bar{r}}_{0}=X_{0},\;{\bar{a}}_{0}=X_{0}-\frac{\pi }{2}n_{0},\;{\bar{\phi }}_{0}=-\frac{\pi }{2},\;{\bar{B}}_{0}={\bar{a}}_{0}{d\omega }_{s},\;\Delta {\bar{\tilde{\omega }}}_{0}=0,\;{\bar{q}}_{0}=0$$

The Jacobian matrix of the averaged system (34) at the equilibrium point is:

(35) $$F_{2}\equiv \frac{{\partial f}_{2\mathrm{av}}}{{\partial x}_{2}}=\left[\begin{array}{cccccc} -\frac{d}{2} & 0 & \frac{1}{{2\omega }_{s}} & -\frac{K_{p}}{{2\omega }_{s}} & 0 & 0\\ 0 & -\frac{d}{2} & 0 & 0 & \frac{1}{{2\omega }_{s}} & 0\\ 0 & 0 & 0 & -K_{I} & 0 & 0\\ \lambda_{a} & 0 & 0 & -\lambda_{a} & 0 & 0\\ 0 & 0 & 0 & 0 & 0 & -K_{\omega }\\ 0 & \frac{\lambda_{q}{\bar{a}}_{0}}{2} & 0 & 0 & 0 & -\lambda_{q} \end{array}\right]$$

The conditions for the above Jacobian matrix to be stable are:

(36) $$\left(d+\frac{K_{p}}{\omega_{s}}\right)\;\left(\frac{d}{2}+\lambda_{a}\right)>\frac{K_{I}}{\omega_{s}},\;d\;\left(\frac{d}{2}+\lambda_{q}\right)>\frac{{\bar{a}}_{0}K_{\omega }}{{2\omega }_{s}}$$

If the feedback system is stable, actual resonant frequency is tuned to the specified resonant frequency since $\Delta {\bar{\tilde{\omega }}}_{0}=0$ is achieved.

### 3.3. Resolution Analysis

Similarly to the previous section, applying the averaging method yields the following steady state covariance equation for (31):

(37) $$0=P_{2}F_{2}^{T}+F_{2}P_{2}+Q_{2}$$

where *F*_2_ is defined in (35) and:

$$Q_{2}=\left[\begin{array}{cccccc} \frac{{\Delta \omega }^{2}\sigma_{p}^{2}}{2} & 0 & 0 & 0 & 0 & -\frac{\Delta \omega \sigma_{p}^{2}\lambda_{q}}{2}\\ 0 & \frac{{\Delta \omega }^{2}\sigma_{p}^{2}}{2} & 0 & 0 & 0 & 0\\ 0 & 0 & 0 & 0 & 0 & 0\\ 0 & 0 & 0 & {\left(\frac{\lambda_{a}\pi }{2}\right)}^{2}\left(0.36\sigma_{p}^{2}\right) & 0 & 0\\ 0 & 0 & 0 & 0 & 0 & 0\\ -\frac{\Delta \omega \sigma_{p}^{2}\lambda_{q}}{2} & 0 & 0 & 0 & 0 & \frac{\lambda_{q}^{2}\sigma_{p}^{2}}{2} \end{array}\right]$$

The standard deviation, which is the averaged resolution of the frequency compensation in the frequency tuning control, is derived as:

(38) $$\sigma_{2}=\sqrt{P_{2}(5,5)}$$

where *P*_2_(5,5) denote (5,5) terms of the numerical solution of (37).

## 4. Application to the Drive Axis of a Dual-Mass Gyroscope

In general, a dual-mass gyroscope has two proof-masses linked to each other by a mechanical beam and is designed to oscillate in anti-phase with the same resonant frequency and amplitude [5]. The Coriolis force acts along opposite directions for each of the two masses, and an external force acts in the same direction, enabling cancellation of the acceleration and other common mode effects. However, because of issues such as the difficulties in the drive axis alignment due to the connecting beam, possibility of unexpected vibration mode, and high production cost induced by high precision process, it has been proposed to remove the connecting beam and have the controller oscillate the two masses in anti-phase with the same resonant frequency and amplitude [6].

To evaluate the control performance, the proposed control algorithms are applied to the drive axis of a dual-mass gyroscope without a mechanical linkage between the proof-masses. The equation of motion for the drive axis of a dual-mass gyroscope is described by two second-order differential equations as follows:

(39) $$\begin{array}{l} {\ddot{x}}_{1}+d_{1}{\dot{x}}_{1}+\omega_{1}^{2}x_{1}=f_{1}\\ {\ddot{x}}_{2}+d_{2}{\dot{x}}_{2}+\omega_{2}^{2}x_{2}=f_{2} \end{array}$$

where subscript 1 and 2 denote the first and the second proof-mass, respectively, *x*_1_, *x*_2_ are the displacements of each proof-mass, *d*_1_, *d*_2_ are normalized damping coefficients, *ω*_1_, *ω*_2_ are natural frequencies, and *f*_1_, *f*_2_ are control inputs.

The first proof-mass is set as the leading resonator, and the second one is set as the following resonator, which precisely traces the oscillation pattern of the leading resonator. The proposed frequency and amplitude controls are applied to each mass. The dual-mass gyroscope parameters are taken from a prototype fabricated at Sejong University, and the parameters are:

(40) $$\begin{array}{l} \omega_{1}=2.30\;\mathrm{KHz},\;\omega_{2}=2.53\;\mathrm{KHz}\\ d_{1}=72.26\;{s\text{ec}}^{-1},\;d_{2}=79.5\;{s\text{ec}}^{-1} \end{array}$$

Considering likely manufacturing errors, 10% error is added to *ω*_1_ for *ω*_2_ calculation, and 10% error is added to *d*_1_ for *d*_2_ calculation. The PSD of the displacement measurement noise is assumed to be $\sigma_{p}^{2}=6.92\times 10^{-24}\;m^{2}/\mathrm{Hz}$. The control law applied to the first proof-mass is frequency tracking and amplitude control and is written as:

(41) $$f_{1}=A_{1}\;\text{cos}\;\theta_{1}$$

where *A*_1_ and *θ*_1_ are calculated from (3) and (4), respectively. The control law for the second proof-mass is frequency tuning and amplitude control. In anti-phase, the second proof-mass should oscillate with the same resonant frequency as the first proof-mass, so the control law (25) is modified as:

(42) $$f_{2}=-A_{2}\;\text{cos}\;\theta_{1}+\Delta \hat{\omega }\;x_{2}$$

where *θ*_1_ is identical to (41) and *A*_2_ and Δ*ω̂* are calculated from (3) and (26).

The control parameters are selected to meet the stability conditions in (18) and (36). Table 1 shows the values of the control parameters used in the simulations. Note that the values in Table 1 are non-dimensionalized based on length 1 μm and time 1/*ω*_1_ (sec).

**Figure 3.**
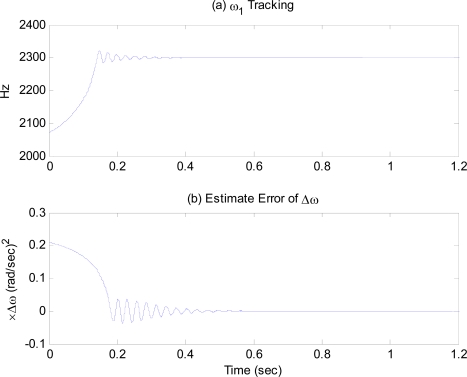
(a) Excitation frequency, (b) Estimation error of the frequency difference.

Figure 3(a) shows that the frequency tracking control on the first proof-mass tracks the resonant frequency. The excitation frequency starting from free oscillation frequency of PLL, *ω*_0_ = 0.9*ω*_1_, reaches the resonant frequency of the proof-mass in 0.4 second and then maintain its value. The averaged resolution of the excitation frequency from (23) is σ_1_ ≈ 0.004 Hz. Figure 3(b) shows the response of the estimation error, Δ*ω̃*, showing that it converges to zero around 0.6 second.

**Figure 4.**
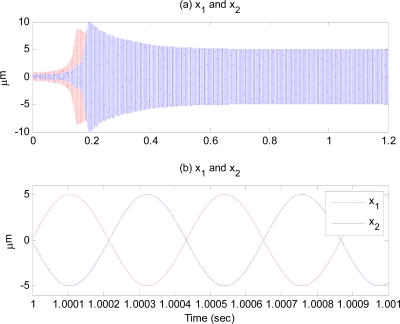
Time response of drive axes.

As soon as the estimation error becomes zero, the dynamic characteristic of the second proof-mass begins to have the same resonant frequency as the first proof-mass, as observed in Figures 4. The estimation error of Δ*ω* calculated from (38), is less than σ_2_ ≈ 0.01% ×Δ*ω̃*. Figure 4(a) is the displacement response of the proof-mass. Its amplitude is maintained at the specified value, *X*_0_ = 5 μm, after some time. Figure 4(b) zooms in the response plotted in Figure 4(a) for 1 msec, showing that the two proof-masses oscillate in anti-phase with same resonant frequency and amplitude.

## 5. Application to Mode Matching for a Vibratory Gyroscope

Most vibratory gyroscopes rely on matching the resonant frequencies of drive and sense axes for high performance. However, manufacturing imperfections result in deviations of the resonant frequencies from their design values. Therefore, various tuning methods have been developed [7,8] and the most commonly employed method is to alter the resonator stiffness by applying bias voltages with dedicated electrodes. In this method, the bias voltage should be maintained under the change in the operating conditions, such as temperature variations.

An alternative mode matching method is presented in this section using the proposed oscillation control algorithms. This method is adaptive to changes in the environment such that if the resonant frequency of sense axis is deviated from that of drive axis, the controller will continually compensate the frequency deviation. Therefore it can be used for on-line implementation.

In this method, the frequency tracking and amplitude control is used in the drive axis, and the frequency tuning control in the sense axis. The amplitude control is not used in the sense axis because the amplitude should be allowed to change according to the input angular rate. Since the drive axis control is same as that in previous section, only the sense axis is considered here. The sense axis of a vibratory gyroscope is modeled as:

(43) $$\ddot{y}+d_{y}\;\dot{y}+\omega_{y}^{2}\;y=-2\Omega \dot{x}+f_{y}$$

where *y* is the sense axis displacement, *ẋ* is the drive axis velocity, *d_y_* is the normalized damping coefficient, *ω_y_* is the natural frequency of sense axis, Ω is the input angular rate about z-axis, and *f_y_* is the control input for sense axis.

Because the frequency tuning control relies on the displacement measurement, it is required to excite the sense axis continually regardless of the presence of an angular rate input. Therefore we propose to insert fictitious angular rate into the sense axis, and the control law (25) is modified as follows:

(44) $$f_{y}={2\Omega }_{\;}\;X_{0}\;\omega_{x}\;\text{cos}\;\theta_{x}+\Delta {\hat{\omega }}_{y}\;y$$

where *ω_x_* and *θ_x_* are drive axis resonant frequency and its integral resulting from drive axis control, respectively. Ω_0_ is a fictitious angular rate, *X*_0_ is the amplitude of drive axis oscillation, and Δ*ω̂_y_* is the estimate of $\left(\omega_{y}^{2}-\omega_{x}^{2}\right)$, which is calculated from (26). The first term in (44) is equal to 2Ω_0_*ẋ* if the drive axis is at resonance using the drive axis control (41). By substituting (44) into (43), and modifying (34) and calculating the averaged equilibrium point yield:

$$\begin{array}{l} {\bar{r}}_{0}=\;a_{0}+\frac{\pi }{2}n_{0},\;{\bar{\phi }}_{0}=-\frac{\pi }{2},\\ a_{0}=\frac{{2X}_{0}\left(\Omega -\Omega_{0}\right)}{d_{y}},\;\Delta {\bar{\tilde{\omega }}}_{0}=0,\;{\bar{q}}_{0}=0 \end{array}$$

where *a*_0_ is the magnitude of sense axis oscillation, which is proportional to input angular rate.

**Figure 5.**
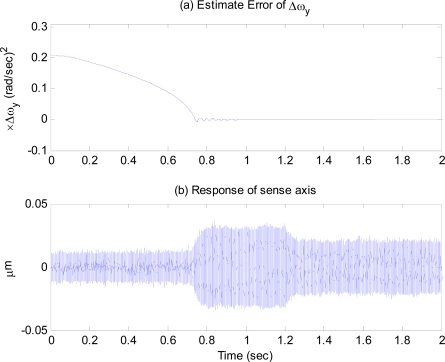
(a) Frequency tuning estimation error, (b) Time response of sense axis.

**Figure 6.**
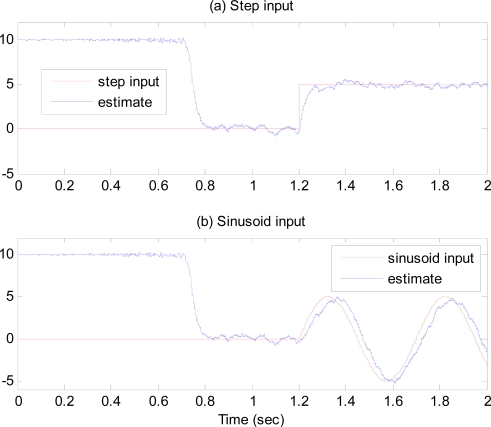
Time response of angular rate estimates to (a) step input, (b) sinusoid input.

Simulations are conducted with the same gyroscope data given by (40), where subscript 1 and 2 are replaced by *x* and *y*. Fictitious input angular rate is assumed to be 10 deg/sec. Figure 5(a) shows the time response of the frequency tuning estimation error. Figure 5(b) shows the time response of the sense axis to a step input angular rate of 5 deg/sec applied at 1.2 sec. It is observed that as soon as the tuning estimation error becomes zero around 0.8 sec, the dynamic characteristic of the sense axis begins to have the same resonant frequency as the drive axis, and the gyroscope is ready to work, with the response of sense axis after 0.8 sec larger than that before. Figure 6 shows the estimates of the angular rate response to step and sinusoidal input angular rates when the modes are matched. These simulation results illustrate that the resonant frequencies of the drive and sense axes are precisely matched, and the gyroscope performance is greatly improved.

## 6. Conclusions

In this paper, two frequency and amplitude control algorithms are presented. One control algorithm excites the resonator at its own resonant frequency, and the other alters the resonator dynamics to place the resonant frequency at a specified frequency which is chosen by the designer. These control algorithms maintain specified amplitude of oscillations. The stability of the entire feedback system was analyzed using the averaging method, and the stability criteria were proposed so that it can be used as guidelines for selecting the control parameters. In addition, the effects of displacement measurement noise on the tracking and estimation of the resonant frequency were analyzed.

In order to evaluate the performance of the proposed control algorithms, we apply them to two important applications. The first one was application to the drive axis control loop for a dual-mass gyroscope without a mechanical linkage between two proof-masses. The second one was application to modal frequency matching in a vibratory gyroscope for high performance operation.

Simulation results agreed well with analytical analysis, and demonstrated the effectiveness of the proposed control algorithms. In addition, both the analytical and simulation studies showed that it is possible to make the two proof-masses of a dual-mass gyroscope without mechanical linkage to oscillate in anti-phase at the same resonant frequency and with the same amplitude. The proposed controller also enables the resonant frequencies of the drive and sense axes to be matched precisely by continual compensation the frequency deviations, which greatly improves the gyroscope performance.

## Figures and Tables

**Table 1.** Non-dimensional values of the control parameters.

**Parameter**	**Value**
*X*_0_, *K_p_*, *K_I_*, *λ_a_*	5, 0.018, 1.5 × 10^−5^, 0.05
*ω*_0_, *K_v_, K_z_, λ_p_*	0.9, 0.5, 1.5 × 10^−4^, 0.5
*K_ω_, λ_q_*	1.5 × 10^−4^, 0.5
